# Gene-Editing and RNA Interference in Treating Hepatitis B: A Review

**DOI:** 10.3390/v15122395

**Published:** 2023-12-08

**Authors:** Nadiia Kasianchuk, Krystyna Dobrowolska, Sofiia Harkava, Andreea Bretcan, Dorota Zarębska-Michaluk, Jerzy Jaroszewicz, Robert Flisiak, Piotr Rzymski

**Affiliations:** 1 Faculty of Biology, Adam Mickiewicz University in Poznań, 61-614 Poznań, Poland; 2 Collegium Medicum, Jan Kochanowski University, 25-317 Kielce, Poland; krystyna.dobrowolska98@gmail.com; 3 Junior Academy of Sciences of Ukraine, Regional Branch in Dnipro, 49000 Dnipro, Ukraine; sofiiaharkava@gmail.com; 4 National College “Ienăchiță Văcărescu”, 130016 Târgoviște, Romania; andreea.bretcan@yahoo.com; 5 Department of Infectious Diseases and Allergology, Jan Kochanowski University, 25-317 Kielce, Poland; dorota1010@tlen.pl; 6 Department of Infectious Diseases and Hepatology, Medical University of Silesia in Katowice, 41-902 Bytom, Poland; jerzy.jr@gmail.com; 7 Department of Infectious Diseases and Hepatology, Medical University of Białystok, 15-540 Białystok, Poland; robert.flisiak1@gmail.com; 8 Department of Environmental Medicine, Poznan University of Medical Sciences, 60-806 Poznań, Poland

**Keywords:** hepatitis B, antivirals, CRISPR/Cas, TALENs, RNA interference, therapeutic vaccinations, mRNA vaccines

## Abstract

The hepatitis B virus (HBV) continues to cause substantial health and economic burdens, and its target of elimination may not be reached in 2030 without further efforts in diagnostics, non-pharmaceutical prevention measures, vaccination, and treatment. Current therapeutic options in chronic HBV, based on interferons and/or nucleos(t)ide analogs, suppress the virus replication but do not eliminate the pathogen and suffer from several constraints. This paper reviews the progress on biotechnological approaches in functional and definitive HBV treatments, including gene-editing tools, i.e., zinc-finger proteins, transcription activator-like effector nucleases, and CRISPR/Cas9, as well as therapeutics based on RNA interference. The advantages and challenges of these approaches are also discussed. Although the safety and efficacy of gene-editing tools in HBV therapies are yet to be demonstrated, they show promise for the revitalization of a much-needed advance in the field and offer viral eradication. Particular hopes are related to CRISPR/Cas9; however, therapeutics employing this system are yet to enter the clinical testing phases. In contrast, a number of candidates based on RNA interference, intending to confer a functional cure, have already been introduced to human studies. However, larger and longer trials are required to assess their efficacy and safety. Considering that prevention is always superior to treatment, it is essential to pursue global efforts in HBV vaccination.

## 1. Introduction

The hepatitis B virus (HBV) is a hepatotropic envelope virus (Hepadnaviridae family, Orthohepadnavirus order) with partially double-stranded, relaxed circular DNA, which is converted in infected hepatocytes to closed, circular DNA (cccDNA) [1]. In addition, HBV DNA can be integrated into the host genome, and these integrates tend to be more stable than cccDNA due to the relatively rapid turnover of the latter [2,3]. HBV continues to cause a substantial health burden, with an estimated global prevalence of 3.2% in 2022 and 257.5 million infected individuals, of which only 14% were diagnosed, and only 8% of those eligible were treated [4,5]. The HBV infection can be clinically presented as acute or chronic hepatitis, and the infection persistence is linked mainly to the immune anergy [6]. The progression to the chronic infection is defined as the presence of serum hepatitis B surface antigen for at least six months, which is accompanied by the disappearance of anti-HBcIgM. On the other hand, during exacerbation of chronic hepatitis B, anti-HBcIgM may reappear. Chronic infection will develop in over 90% of infants infected with HBV during the perinatal period, in 25–50% of children infected at the age of 1–5 years, and in 6–10% of older individuals, including adults [7]. Chronically infected individuals are an important source of HBV transmission as they can be asymptomatic and unaware of their status. In the long term, chronic HBV infection increases the risk of liver cirrhosis and hepatocellular carcinoma (HCC). One-fifth of patients may even experience extrahepatic manifestations such as cryoglobulinemic vasculitis, glomerulonephritis, hearing loss, non-rheumatoid arthritis, non-Hodgkin lymphoma, or polyarteritis nodosa [8,9]. Available data indicate that 55% of mortality due to primary liver cancer and 45% of deaths from liver dysfunction are attributable to HBV infection [10,11]. In 2019, chronic hepatitis, cirrhosis, and liver cancer contributed 9%, 59%, and 32% of disability-adjusted life years (DALYs) caused by HBV, respectively. At the same time, HBV is responsible for 18.2 million DALYs globally and for 0.7% of all global DALYs [12].

The prevalence of HBV is geographically diversified. African and East Asian regions are considered endemic due to a prevalence above 8%, Eastern Europe and the Mediterranean Basin are moderately endemic (2–8% prevalence), whereas Western Europe and North America have a low endemicity (<2% prevalence) [4,13,14,15]. The vast majority of global HBV infections are caused by five out of ten known genotypes (A–J), with genotype C being the most common (26% of all infections), followed by genotypes D (22%), E (18%), A (17%), and B (14%) [16]. The prevalence of HBV genotypes also varies by world region, e.g., B and C are more frequent in Asia, while A, D, and E are predominant in sub-Saharan Africa [16,17]. However, the distribution of genotypes can also significantly vary in local terms, e.g., within a particular country [18,19]. There is evidence that they differ in mutational rate, modes of transmission (perinatal/vertical or horizontal), the tendency of chronicity, seroconversion rate, serum HBV DNA levels, histological activity, and clinical outcomes in terms of liver cirrhosis and HCC [20]. The outcomes of HBV infection can be further complicated under co-infection with the hepatitis delta virus (HDV), a small, defective RNA virus that lacks its envelope, requires the HBsAg PreS-1 domain of L-HBsAg for its assembly, and whose transmission depends on contaminant presence of HBV [21,22,23].

**Figure 1:**
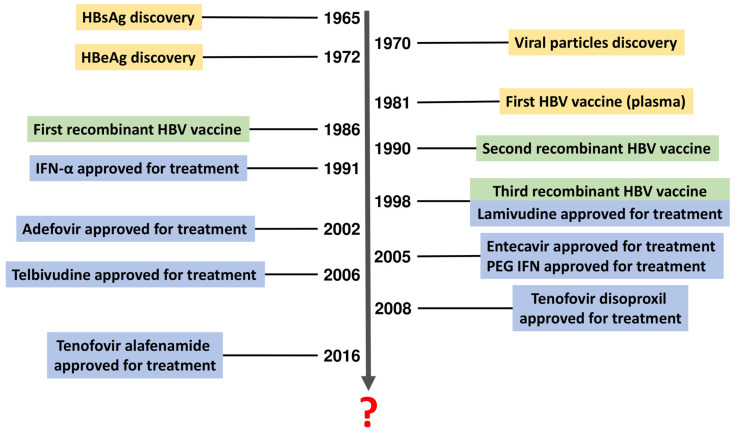
History of discoveries related to hepatitis B virus and hepatitis B prevention and treatment. Data of approval refer to decisions by the U.S. Food and Drug Administration.

The HBV infection can be effectively prevented with vaccination within 24 h of birth, followed by 2–3 additional doses. The first HBV vaccine was authorized in 1981, and in the 1986–1998 period, three generations of recombinant vaccines were subsequently introduced (Figure 1). HBV vaccination is a proven life-saving intervention, decreasing the occurrence of HBV infections, and is estimated to avert 38 million deaths over the lifetime of persons born between 2000 and 2030 in 98 low- and middle-income countries [24]. It is also economically justified. For example, in the 2001–2020 period, it saved USD 49 billion in cost of illness and USD 81 billion in total economic and societal values in 73 low- and middle-income countries [25].

However, due to vaccine hesitancy as well as challenges in vaccine availability in some regions, more than half of the world’s infants in 2021 did not receive the first dose within 24 h of birth, and over 1.5 million preventable new cases of HBV continue to occur each year [26]. According to the World Health Organization, hepatitis B elimination requires reaching a level of 90% vaccination coverage in infants by 2030 [27]. Likely, many countries will not achieve this target in the given timeframe [4,28].

Treatment of hepatitis B infection also remains challenging as there is currently no definitive cure in the case of patients chronically affected by the virus. So far, two main types of treatment have been approved, interferons (IFNs) and nucleos(t)ide analogs (NAs) (Figure 1), both of which do not clear cccDNA persisting in hepatocytes. Instead, available therapies target HBV DNA suppression, HBeAg and HBsAg loss/seroconversion, and normalization of alanine aminotransferase (ALT) levels to prevent disease progression and development of HCC and improve patient-oriented outcomes, including survival and quality of life. However, these therapies are far from optimal. IFN therapies are associated with a wide range of adverse events, while some patients do not reach a functional cure (defined as sustained undetectable circulating HBsAg and HBV DNA after a finite course of treatment) [29]. The use of NAs is associated with a better safety profile and deeper suppression of viral replication, but the long-term outcomes, including HBsAg loss and seroconversion to anti-HBs antibodies, are far from satisfactory [30]. Despite numerous clinical trials, since 2016, when tenofovir alafenamide was authorized by the U.S. Food and Drug Administration, no novel hepatitis B therapies have been approved (Figure 1). In 2020, bulevirtide received conditional marketing authorization in the European Union (changed to full marketing approval in 2023) for use in HBV patients with compensated liver disease who are co-infected with HDV [31]. Its mechanism of action includes blocking sodium taurocholate co-transporting polypeptide, a bile salt liver transporter known to be an essential receptor for HBV/HDV entry [32]. Therefore, bulevirtide may potentially be beneficial for HBV treatment, although its approval (not granted by the U.S. Food and Drug Administration [33]) only concerns patients co-infected with HBV [31]. Despite the lack of new therapies, some improvement has been made with regard to existing therapy optimizations, including a better understanding of sequential or combination use of pegylated IFN and NAs.

Therefore, unsurprisingly, it is repeatedly highlighted that further efforts in diagnosis, prophylaxis, and treatment are needed to reach ambitious and much-needed HBV elimination goals. Without more significant investments in different interventions, mortality due to hepatitis B will cost the global economy an estimated USD 780 billion between 2022 and 2050. Over the recent decades, increased hopes of medicine have been related to biotechnology and its rapidly evolving tools, such as gene editing, immunotherapies, or novel vaccine platforms [34,35,36]. Some of these approaches have already found their place in the field of viral infectious diseases, with a recent example of mRNA and adenoviral vector vaccines against COVID-19, which played a role in decreasing the SARS-CoV-2 burden, particularly in terms of decreasing disease severity and averting deaths [37,38,39,40]. In 2022, the clinical trial of EBT-101, a therapeutic candidate based on the adeno-associated virus delivering CRISPR/Cas9 guide RNAs to excise large portions of the HIV genome from host cells, was initiated [41]. Therefore, developing biotechnology-based strategies for treating chronic HBV infection appears reasonable as a part of global efforts to eliminate this virus.

The genetic diversity of chronic HBV infection is driven by the poor proof-reading activity of the viral polymerase and rapid turnover of active cccDNA. This causes the evolution of thousands of co-existing genetic variants within an individual host and the rapid evolution of new variants and enrichment of escape variants under selection pressure. This feature of chronic HBV infection is a critical stumbling block for any sequence-dependent oligonucleotide approach, which includes CRISPR Cas9, siRNA, antisense and immunological approaches such as engineered T-cells, engineered antibodies, therapeutic vaccines, and even birth dose vaccination [42,43,44,45,46,47]. Unfortunately, the COVID-19 pandemic adversely affected the development of novel therapeutic candidates and the initiation of clinical trials outside the SARS-CoV-2 realm [48,49]. However, in May 2023, the World Health Organization declared that COVID-19 is no longer a Public Health Emergency of International Concern [50], raising hopes of revitalizing advances in other medical fields, including viral hepatitis. The present paper reviews the current progress in biotechnological approaches in functional and definitive HBV treatments, and discusses the advantages and challenges of tools such as gene-editing tools (zinc fingers, transcription activator-like effector nucleases (TALENs), and CRISPR/Cas9) and RNA interference.

## 2. Current Options in HBV Treatment

Regardless of HBe status, indications for treatment are based on a combination of three main criteria: serum HBV DNA level, serum ALT level, and severity of liver disease [29,51]. According to the current European Association for the Study of the Liver (EASL) guidelines, all patients with HBeAg-positive or -negative chronic hepatitis B, defined by HBV DNA > 2000 IU/mL, elevated ALT activity, and/or at least moderate liver necroinflammation or fibrosis, should be treated. Patients with compensated or decompensated cirrhosis with any detectable HBV DNA load require treatment, regardless of ALT activity [29,51]. According to the recommendations of the American Association for the Study of Liver Diseases (AASLD), antiviral therapy should be initiated in patients with elevated ALT activity > 2 ULN or the presence of significant histological disease and elevated HBV DNA above 2000 IU/mL in HBeAg negative patients or 20,000 IU/mL in HBeAg positive patients; however, it stipulates that in patients with significant fibrosis/cirrhosis, treatment is recommended regardless of ALT levels [52]. The most recent update of Chinese guidelines for chronic hepatitis B recommends the expansion of antiviral treatment to all individuals with detectable HBV-DNA and ALT > ULN or with conditions suggestive of a higher possibility of HBV-related complication, including those over 30 years old. This is a significant shift in the paradigm of HBV treatment, although its long-term clinical effects, as well as feasibility, are yet to be seen [53]. In 2024, updates of WHO, EASL, and AASLD guidelines for treating hepatitis B are planned to be released.

Importantly, patients with chronic HBV infection, who are cirrhotic, with extrahepatic symptoms and a family history of HCC, can be treated, even when the typical indications for treatment are not met. Moreover, individuals with HBV DNA > 20,000 IU/mL and sustained ALT activity at the level exceeding two-fold the upper reference threshold should begin treatment regardless of the degree of liver fibrosis [29]. The available therapies include monotherapy with pegylated IFN alpha 2a (PEG-IFN-α2a) with a finite duration of 48 weeks or treatment with NAs with long-term duration.

### 2.1. Pegylated Interferon- α2a (PEG-IFN-α2a) in Chronic Hepatitis B Treatment

PEG-IFN-α2a induces long-lasting immunological control of HBV, with HBsAg loss achieved in 30–50% of patients at long-term follow-up [54]. It inhibits HBV replication by reducing RNA transcription. This effect is achieved by cccDNA-bound histone hypoacetylation and cccDNA transcriptional co-repressor active recruitment [55,56]. PegIFN is indicated for the treatment of patients with chronic hepatitis B and compensated liver disease due to HBV and is the current standard of care for the treatment of HBV/HDV co-infection in patients with this stage of liver disease.

In addition to a patient’s immune characteristics and phase of HBV infection, an important factor influencing the chances of response to antiviral IFN therapy is the HBV genotype. Compared with B–D, genotype A is associated with significantly higher rates of HBeAg and HBsAg loss during IFN therapy [52]. Despite an overall higher effect on HBsAg levels than NA, its use is limited by an uncomfortable route of administration in the form of subcutaneous injections administered once weekly, contraindications, and side effects [51,54]. Severe comorbidities, decompensation of liver cirrhosis, neutropenia, and thrombocytopenia are contraindications to using PEG-IFN-α2a [54]. The most common side effects during IFN therapy are mild flu-like symptoms, headache, and fatigue, but more severe symptoms such as neutropenia or psychiatric, neurological, and endocrine disorders were also reported [55]. It is worth noting that the safety profile of pegIFN is much more favorable in patients with HBV infection compared to HCV, and that hematologic abnormalities such as thrombocytopenia and leukopenia are generally laboratory findings and are rarely symptomatic, except in patients with advanced liver disease.

Patients treated with PEG-IFN-α2a should be monitored both for tolerability and effectiveness of therapy, defined as a reduction in HBV DNA viral load of at least two log10 at week 12 of treatment and a value below 2000 IU/mL at the end of, and six months after, therapy completion. A sustained virological response is considered when the viral load remains below 2000 IU/mL for at least 12 months after PEG-IFN-α2a therapy is completed. Serological response to treatment is characterized by the HBeAg loss and HBe seroconversion in HBeAg-positive patients and HBsAg loss and HBs seroconversion in all HBV-infected patients. The biochemical response to PEG-IFN-α2a treatment is defined as the normalization of ALT activity. It requires long-term monitoring because some patients may experience transient increases in ALT levels, especially during the first year after treatment, before long-term biochemical remission. The response to PEG-IFN-α2a treatment defined in the above-mentioned ways allows the application of the early stopping rules. When the HBV DNA level does not decrease after 12 weeks, treatment should be discontinued, and therapy with NAs should be initiated, preferably with entecavir (ETV), tenofovir disoproxil fumarate (TDF), or tenofovir alafenamide (TAF), as they reveal low risk of resistance [29,51].

### 2.2. Nucleos(t)ide Analogs (NAs) in Chronic Hepatitis B Treatment

NAs block the HBV DNA polymerase enzyme, thus inhibiting the virus replication and further viral spread [57]. NAs strongly suppress HBV replication, but their effect on HBsAg levels is low, especially in HBeAg-negative patients [58]. Therefore, they require long-term, often lifetime use in most patients. However, the convenient form of oral use and an excellent safety profile do not translate into significant challenges with non-adherence. Potent NAs with a high barrier to resistance (entecavir, tenofovir disoproxil fumarate, and tenofovir alafenamide) are the treatment of choice regardless of the severity of liver disease. Moreover, they are the only treatment option for several patient subpopulations, including those with decompensated cirrhosis, after liver transplantation, or those with severe chronic hepatitis B exacerbation. However, the long duration of treatment based on NAs increases the risk of developing resistance. This is primarily a significant issue for low-barrier analogs such as lamivudine and adefovir [59]. So far, a number of studies have shown that despite long-term treatment, the probability of developing resistance to strong NAs, i.e., entecavir and tenofovir, remains low [29,60]. However, mutations decreasing susceptibility to these therapeutics were identified, and as indicated, resistant mutants could emerge due to the accumulation of multiple mutations during long-term use, especially in HBeAg-negative individuals [61].

Treatment with NAs is considered effective if HBV replication is suppressed below the threshold of detection in serum. Suppression of viremia is usually accompanied by biochemical normalization and histologic improvement [29,51,60,62,63]. Treatment failure may be categorized into (i) primary non-response, defined as a decrease in serum HBV DNA of less than one log10 IU/mL after three months of treatment; (ii) partial virological response, defined as a decrease in HBV DNA of more than one log10 IU/mL but detectable after at least 12 months of therapy; and (iii) virological breakthrough, which is a confirmed increase in the HBV DNA level of more than 1 log10 IU/mL compared to the lowest HBV DNA value on-therapy. One should note that suppression of HBV replication below the threshold of detection in serum is not always necessarily equal to its total elimination.

The 5-year cumulative probability of virological response and HBe loss in HBeAg-positive chronic hepatitis B patients participating in registration trials with ETV was 99% and 53%, respectively, while HBe-negative patients achieved a virological response of 98% [29]. For HBe-positive patients treated in clinical trials with TDF, the cumulative 10-year values were 100% for virological response and 52% for HBe loss [64]. In the HBe-negative population, 98% of patients achieved a virological response after ten years of TDF therapy [64]. TAF therapy showed no inferiority to TDF treatment with a better kidney and bone safety profile [63]. The high virological response rates to ETV and TDF therapies from clinical trials have been confirmed by real-world analyses [60,65,66].

The optimal endpoint of NA treatment, regardless of baseline HBe status, is HBsAg loss with or without anti-HBs seroconversion, which is very rare. No HBeAg-negative patient cleared HBsAg during the first year of ETV or TDF therapy, and about 1% achieved this endpoint during long-term therapy [62]. Promising effects of increasing the rate of HBsAg loss in patients were obtained from a combination or sequential therapy consisting of TDF and PEG-IFN-α2a, although this strategy is currently not included in clinical recommendations [67].

According to recommendations, treatment discontinuation is acceptable in HBe-negative patients who have achieved HBV DNA suppression lasting at least three years, and for HBeAg-positive patients who have achieved stable HBeAg seroconversion and undetectable HBV DNA after at least one year of consolidation [29]. Such an approach should be limited to patients without advanced liver fibrosis, and close monitoring after treatment discontinuation is obligatory, keeping in mind that the risk of virological relapse remains high [68,69]. In an international, multicenter, and multiethnic study—RETRACT-B—based in Europe, Asia, and North America of 1552 HBe-negative, virally suppressed patients who stopped NA therapy, 83.4% had virological relapse. NA withdrawal was more beneficial for white patients with HBsAg levels < 1000 IU/mL and Asians with HBsAg levels < 100 IU/mL, regardless of their HBeAg status at the start of the therapy [70]. In another multicenter study, 166 HBeAg-negative patients with HBV DNA < 172 IU/mL treated with NA for at least 4 years were randomly assigned to either stop or continue therapy and were observed for 96 weeks. Treatment cessation was associated with significantly higher rates of HBsAg loss at week 96 in the group that ceased NA use compared to those continuing therapy (10.1% vs. 0%, respectively). In this instance, a minority of patients were in need of retreatment (13.9%, 11/79) [71]. In a prospective study, 42 non-cirrhotic HBeAg-negative patients were randomly assigned to stop or continue monotherapy with TDF after over four years of treatment. Of 13 patients who remained off-therapy to week 144, 43% achieved HBsAg loss or HBV DNA < 2000 IU/mL, which offers a potential for long-term HBV suppression after stopping NA therapy even after initial fluctuations in viral load and ALT levels [72]. The risk of virological relapse is higher in patients infected with genotype D and treated with NAs with low barriers of resistance, and determining with high accuracy those more susceptible to it remains the topic of discussion [73]. To date, the most reliable biomarker for forecasting SVR is quantitative HBs assessment; rapid HBsAg decline during NA therapy helps identify patients with a higher possibility of achieving functional cure [74]. Nevertheless, it is worth mentioning that HBsAg decline is not necessarily linear, and after some level of decline, a plateau in its concentration is often seen. Therefore, the predictive value of the HBsAg level as a single marker declines and is not an established part of PEG-IFN therapy. HBV core-related antigen (HBcrAg), which remains present in serum even with undetectable HBV DNA and after loss of HBsAg, can be used to predict HBV reactivation after cessation of NA treatment [75,76,77]. Similarly to HBcrAg, HBV RNA is a sensitive biomarker of cccDNA transcription; NAs cause suppression of HBV DNA but do not affect HBV RNA levels [78].

## 3. Biotechnological Approach to HBV Treatment

### 3.1. Gene-Editing Tools

**Figure 2:**
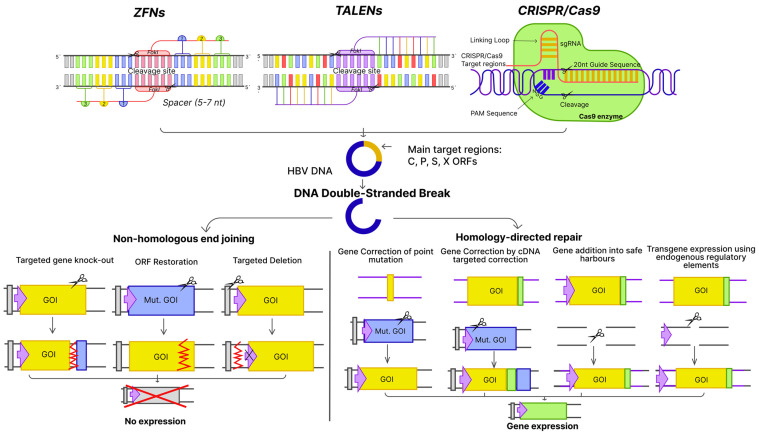
Schematic representation of the mechanism of action of different gene-editing tools, zinc-finger nucleases (ZFNs), transcription activator-like effector nucleases (TALENs), and the clustered regularly interspaced palindromic repeat-Cas system (CRISPR/Cas), in relation to HBV infection. Graph created using Figma.

Neither therapy authorized so far, i.e., PEG-IFN-α2a and NAs, affect HBV cccDNA that exists episomally and is responsible for viral persistence and reactivation. It has been reported that a few copies of cccDNA may be sufficient for infection rebound after cessation of treatment. A complete cure of HBV would only be possible through clearance of cccDNA. In addition, HBV DNA can be integrated with the host genome, a phenomenon that likely contributes to the risk of HCC [79]. Some studies suggest that potentially functional HBx protein, which is required for viral replication, can be produced from integrated HBV DNA [80,81]. Therefore, therapies aiming simultaneously at cccDNA and integrated HBV elimination might be needed for the sterilizing cure in some cases. Reaching such goals will only be possible with gene editing. The advances in the biotechnology field have resulted in the development of tools that have the potential for use in definitive HBV treatment, i.e., zinc-finger proteins, transcription activator-like effector nucleases (TALENs), and the clustered regularly interspaced palindromic repeat-Cas system (CRISPR/Cas9) (Figure 2). Their potential application in the treatment of HBV infections is reviewed in the subsequent subsections.

However, one should note that employing gene-editing tools, regardless of their generation, may not always be a sufficient approach due to the potentially broad spectrum of cells that HBV can infect. Although it was identified that HBV requires a sodium taurocholate co-transporting polypeptide, a liver-specific bile acid transporter, as an essential receptor for cellular entry [82,83], it was recently discovered that a fraction of its intact virions can be released in exosomes, extracellular vesicles that can travel to distant tissues, and through which HBV can gain access to a wide range of cells previously considered as non-permissive, such as endothelial cells or neurons [84,85]. Although such events may be rare in HBV-infected individuals, they may potentially decrease the effectiveness of gene-editing tools employed for treatment—an issue requiring further research.

There are also some general challenges of gene-editing tools that need to be considered. Firstly, they require the employment of an appropriate delivery system, which can be either based on a viral vector or a non-viral vehicle. The former reveals high delivery efficiency, but their immunogenicity and potential insertional mutagenesis are major obstacles and the cause of safety concerns. The latter are inert to host genetic material, but the efficiency of their delivery to specific target cells can be challenging [86,87]. Secondly, gene-editing tools may also induce off-target editing, i.e., nonspecific and unintended genetic modifications [88]. The functional significance of such off-targeting depends on the region in which they occur, urging the need to monitor this phenomenon in studies of the safety of gene-editing applications and long-term safety monitoring.

#### 3.1.1. Zinc-Finger Proteins (ZFPs)

Zinc-finger proteins (ZFPs) are a diverse family of transcription factors that play crucial roles in development, gene regulation, and DNA repair. These proteins typically contain one or more zinc-finger motifs, each comprising a zinc ion coordinated by cysteine and histidine residues. The specific DNA-binding specificity of ZFPs is determined by the amino acid sequence of the zinc-finger domain [89]. On the other hand, zinc-finger antiviral proteins (ZAPs) are a specific subset of ZFPs that are specialized immune proteins and function as host defense mechanisms against viral infections. ZAPs contain a unique set of zinc-finger motifs and other domains that enable them to specifically recognize and degrade viral RNA, thereby preventing viral replication. Their role in antiviral immunity sets them apart from other zinc-finger proteins, as their primary function is to combat viral infections [90,91,92].

Zinc-finger nucleases (ZFNs) are yet another class of ZFPs that are engineered to combine the DNA-binding specificity of ZFPs with the DNA-cleavage activity of nucleases (Figure 2) and, as a result, have a specific purpose in genome editing [93,94]. ZFNs have been pioneered as tools for introducing site-specific double-strand breaks in desired genomic locations [95]. At their core, ZFNs are synthetic entities crafted by coupling multiple zinc-finger (ZF) domains to the cleavage module of the FokI type IIS restriction endonuclease. Each ZF domain, spanning approximately 30 amino acids, secures a zinc atom through coordination involving two cysteine and two histidine residues. An integral alpha-helix within each ZF domain is tailored to discern a distinct DNA triplet. Consequently, by chaining several such ZF domains, ZFNs can discern extended DNA sequences [96]. For the cutting action to occur, the FokI nuclease domain must dimerize when two clusters of ZF domains are appropriately juxtaposed and oriented (Figure 2). Decoupling DNA-binding and cleaving functionalities in ZFNs is advantageous, allowing for isolated optimization to enhance the cleaving efficiency for specifically chosen targets.

ZFNs have shown potential for use in curing HBV infection, and a number of *in vitro* and *in vivo* attempts to employ them for this purpose have been made, a summary of which can be found in Supplementary Table S1. One of the first attempts involved an *in vitro* model and duck HBV, which is a model virus for human HBV, and, as shown, the designed ZF proteins were successfully binding to the viral enhancer and interfering with the viral transcription, ultimately leading to decreased emergence of viral products [97]. As demonstrated, ZNF, especially the pair targeting only the viral polymerase-encoding open reading frame, could effectively disrupt HBV DNA sequences and substantially suppress HBV replication [98]. This suppression was notably pronounced when ZFNs were delivered using scAAV vectors, highlighting their antiviral capabilities in HepAD38 cells over an extended period. Another study highlighted a ZNF pair targeting the polyadenylation site near the N-terminus of the core protein. This construct cleaved both homo- and heterodimeric targets, with a significant portion (26%) of DNA remaining linear three days post-transfection [99]. On the other hand, the disruption of cccDNA either did not show a positive reproducible outcome or was not tested at all. Meanwhile, a ZNF pair targeting both polymerase- and core protein-encoding open reading frames resulted in the lowest performance and the highest cytotoxicity. Additionally, the aforementioned ZNF pair had the highest potential for off-target effects due to its shorter 15 nt recognition sequence, as opposed to the typical 18 nt [98].

In summary, the ZF-based tools for hepatitis B treatment show some promise in HBV suppression and inactivation but are far from being ready to use in humans since they were mostly tested in *in vitro* experimental models, with only a few *in vivo* studies (Table S1). According to the available evidence, off-targets at the target site do not appear to be a concern, but their occurrence in other regions of the genome was usually not studied, encouraging further studies in this regard. Importantly, cytotoxicity of ZF-based tools was reported in some *in vitro* models and potential effects on host gene expression, indicating the need for more safety testing using *in vivo* models.

ZAPs, not being gene-editing tools directly, still possess unique zinc-finger motifs and other domains that enable them to recognize and degrade viral RNA, thereby impeding viral replication. These findings have raised the prospect of utilizing ZAPs as a component of anti-HBV therapeutic strategies [100,101]. Overexpression of both human ZAP and the N-terminus of rat ZAP significantly reduced HBV DNA replication in various cell lines, primarily through a decrease in viral pgRNA levels. Interestingly, the N-terminal 254 amino acids of human ZAP displayed an anti-HBV function similar to full-length ZAP, suggesting that the N-terminal region of ZAP is crucial for its antiviral activity against HBV. Transgenic mouse models were also employed to assess the potential therapeutic impact of ZAP against HBV. The results showed reduced HBV RNA levels and a marked suppression of HBV DNA replication. Additionally, ZAP’s inhibitory effect extended to viral protein expression, as indicated by fewer HBsAg-positive and HBcAg-positive cells in liver tissue [100].

In summary, ZFNs exhibited a promising performance in reducing HBV replication and interfering with viral transcription, particularly in early studies. However, although initially explored, the interest in their application was largely replaced by a focus on more efficient gene-editing technologies such as TALENs and CRISPR due to their greater precision and versatility.

#### 3.1.2. Transcription Activator-like Effector Nucleases (TALENs)

TALENs have emerged as a powerful genome-editing tool, offering an alternative to ZNFs. Originating from the plant pathogenic bacteria of the *Xanthomonas* genus, TALENs function as dimers, utilizing the nonspecific FokI domain for DNA cleavage [102,103]. Structurally, a TALEN unit comprises a central DNA-binding domain consisting of 12–28 tandem repeats built of 33–35 amino acids, each recognizing a single nucleotide, providing a clear protein–DNA interaction code (Figure 2). The amino acid at position 13 uniquely recognizes a nucleotide in the DNA’s major groove, with stability provided by the amino acid at position 12 [104,105]. Compared to ZNFs, TALENs showed fewer off-target effects and cytotoxicity, combined with high specificity and versatility [106]. Such characteristics have led to numerous applications of TALENs across organisms, including studies on their potential role in treating chronic viral diseases, including HBV infection [107,108,109]. Furthermore, TALEN-driven mutagenesis is not delayed by heterochromatin in contrast to CRISPR/Cas9 [110].

Studies involving cell cultures and animal models have consistently highlighted the effectiveness of TALENs in suppressing HBV replication. Their summary is provided in the Supplementary Table S2. As shown, S-TALEN resulted in an *in vivo* reduction in HBsAg levels exceeding 90% [111]. Furthermore, interventions with both S- and C-TALEN induced mutations in a substantial proportion of HBV sequences, markedly more than in control groups, underscoring a robust antiviral effect [111]. This efficacy aligns with findings from other research, which reported pronounced reductions in both HBsAg and HBeAg across diverse HBV genotypes and TALENs’ generations [112,113]. Several articles have also highlighted a significant (70–90%) reduction in Viral Particle Equivalents *in vivo* under core and surface open reading frames targeting TALENs [111,113]. The therapeutic potential of TALENs was further augmented when paired with established therapeutic agents such as IFN-α [112] or when combined with artificial antiviral primary miRNAs [114].

Nevertheless, some challenges persisted, such as the time-dependency of TALENs’ efficacy [111,113], necessitating further research to determine optimal expression duration. Additionally, despite the evident potential of TALEN treatments, their effectiveness has not consistently manifested across all research settings. Specifically, both the first-generation TALENs and the third-generation TALENs with Sharkey mutations, targeting the core open reading frame, left HBsAg secretion unaffected [113]. Moreover, while TALENs L3/R3 targeted sites near those of L2/R2, they exhibited little to no effect on HBeAg or HBsAg production, suggesting that even slight variations in target sites can dramatically impact TALENs’ antiviral efficiency [112]. Such inconsistencies in results emphasize the need for further refinements and a deeper understanding of the factors influencing TALENs’ therapeutic outcomes.

Another crucial target in this endeavor is cccDNA [113] because it acts as a persistent intrahepatic viral reservoir, facilitating the chronic virus nature and allowing for viral resurgence after treatment is halted [115]. However, due to its unique circular configuration and stable nature within hepatocyte nuclei, cccDNA is a more challenging substrate for targeted mutation than plasmid DNA or integrated DNA [116]. Bloom et al. reported the successful targeted mutagenesis, boosted by hypothermic conditions and triple TALENs transfection, resulting in the disruptions of approximately 35% of cccDNA molecules [111]. Another study also confirmed a decrease in cccDNA by 10–20% in Huh7 cells treated with TALENs targeting the P open reading frame and two-fold in TALENs targeting the C open reading frame [112]. It is worth noting that mouse hepatocytes do not facilitate cccDNA formation, which restricts the ability to explore this aspect *in vivo* [113]. In conclusion, TALENs offer promise in combating chronic HBV infections, demonstrating high specificity and effectiveness in cell and animal models. However, challenges in optimizing their efficacy and targeting cccDNA persist, warranting further research.

#### 3.1.3. The Clustered Regularly Interspaced Palindromic Repeat-Cas System (CRISPR/Cas)

CRISPR-Cas is a third-generation gene-editing technology that, unlike zinc-finger nucleases and transcription activator-like effector nucleases that employ proteins to target DNA strands, directs the Cas protein to a specific genome location through guide RNA molecules [117,118]. This approach results in improved accuracy and efficiency of applicability of gene-editing and widens its applicability [119]. An increased number of CRISPR-based therapies are entering clinical trials in the fields of bacterial infections, hematological diseases, cancer, metabolic disorders, and genetic conditions [118]. In 2023, the Medicines and Healthcare Products Regulatory Agency in United Kingdon became the first regulatory agency in the world to approve CRISPR-based technology in human treatment, i.e., in sickle cell disease and β-thalassemia [120]. There is also an ongoing interest in exploring the use of CRISPR in viral infections, particularly those that can progress into a persistent state, including HBV (Figure 2) [121,122,123]. The overview of research involving CRISPR/Cas in chronic hepatitis B is given in Supplementary Table S3.

Regarding HBV eradication, the pivotal factor in the system’s efficacy lies in the sgRNAs selected to target specific HBV genomic regions. It is crucial to underscore that, to date, the literature lacks consensus regarding which target is optimal. The initial research in this regard was conducted in 2014, revealing significant promise for applying CRISPR technology in HBV treatment, both *in vitro* and *in vivo*. Several gRNAs demonstrated efficient suppression of HBV proteins, achieving inhibition rates ranging from 20% to 70%. Additionally, vectors containing P1, XCp, and PS2 have substantially reduced HBsAg levels, with inhibition percentages of 16%, 36%, and 64%, respectively [124]. In a further study, the utilization of pCas9, designed to target the X gene, has demonstrated remarkable effectiveness in reducing HBV cccDNA levels, replication intermediates, HBsAg, and HBcAg. Notably, pCas9 targeting the S gene has also shown a significant decrease in the above-mentioned biomarkers, although its overall performance was inferior to that of the X gene targeting [125]. In the recent preprint, targeting of the X gene also led to considerable results, showing the transfection efficiency of approximately 60% and a noteworthy decrease in HBsAg (4.5-fold 96 h post-transfection) and HBV cccDNA levels. Interestingly, in another study, gRNAs designed to target X and C genes displayed lower performance than those targeting P and S ORFs with less than 50% base-editing efficiency [126]. This observation underscores the complexity and inconsistency of CRISPR/Cas9-mediated editing, even when targeting the same gene, emphasizing the need for further investigation into potential variations in outcomes, especially when gRNA binding sites are in close proximity.

CRISPR’s potential to be used against HBV was further corroborated in *in vivo* transgenic mice models. AAV8-delivered CRISPR/SaCas9 treatment exhibited remarkable efficacy in inhibiting HBV-related parameters in mouse models. Expression of the HBV core protein in mouse liver was significantly reduced, corroborating the antiviral impact of the treatment. Serum HBsAg levels were also substantially inhibited, with gRNA T2 also showing significant suppression of HBeAg [127]. Several other independent studies have supported the efficacy of CRISPR-based interventions *in vivo*, consistently showing a significant reduction in serum HBsAg and HBV DNA [124,125,128].

Unlike previous successes, the research conducted by Stone et al. (2021) encountered challenges and provided valuable insights into the intricacies of employing CRISPR technology to combat chronic HBV infections [129]. Despite the application of CRISPR-based therapy, mice continued to exhibit substantial viral loads, and eventually, no complete clearance was achieved. Furthermore, widespread HBV replication was observed in both control and treatment groups without apparent differences. The reductions in total HBV DNA and cccDNA in AAV-SaCas9-treated animals also did not reach statistical significance. Furthermore, even the studies with positive outcomes experienced several important limitations that should be encountered in order to develop effective gene therapies against HBV. An important consideration arises from studies utilizing mouse models, as they do not fully replicate the complexity of HBV infection observed in humans. Specifically, these models lack cccDNA, a pivotal component of human chronic HBV infections [130]. Though generally rare, off-target effects still present a concern in some studies. The potential for unforeseen genetic alterations due to base editing, even at low rates, underscores the need for thorough safety assessments [131]. Long-term safety observations in clinical trials should precede the potential use of such tools in humans. One should also note that delivery of CRISPR/Cas9 specifically to hepatocytes may be challenging [132]. Moreover, point mutations in the HBV DNA region targeted by any guide RNA are likely to abolish Cas9 activity. Moreover, mutagenesis driven by CRISPR/Cas9 can be delayed by heterochromatin, particularly when Cas9 exposure is brief or its intracellular expression is low [133].

Overall, CRISPR-Cas technology offers promising avenues for addressing chronic HBV infections. Nevertheless, given the existing constraints and complexities, further investigation and refinement are essential before transitioning these findings into clinical applications.

### 3.2. RNA Interference Therapeutics

Drugs based on RNA interference (RNAi) emerge from the breakthrough discovery of a fundamental cellular mechanism for silencing gene expression and employ small RNA molecules to reduce the expression of pathological proteins. The specificity of RNAi is a key characteristic of this technology and can be theoretically applicable to modulate any class of molecular targets [134]. The first RNAi-based therapeutic approved by the U.S. Food and Drug Administration was patisiran, which was authorized in 2018 for treating polyneuropathy in people with hereditary transthyretin-mediated amyloidosis [135]. Numerous other candidates employing RNAi are currently in clinical trials, and there is also an interest in using this technology as a novel class of therapeutics in chronic viral infections, including chronic hepatitis B [136,137].

In contrast to the gene-editing approach, candidates based on RNAi are aiming to provide a functional cure instead of complete viral eradication. Moreover, contrary to gene-editing tools, numerous RNAi candidates against HBV have already been introduced into clinical trials. Their summary, with main targets, key findings, and observed challenges, is provided in Table 1. The majority of them were designed for subcutaneous injections (AB-729, VIR-2218, JNJ-3989), ARC-521, bepirovirsen, and RG6346), and, less often, intravenous administration (ARC-520, ARB-1467 and ARC-521). Initially, it was thought that their ineffectiveness was due to their lack of targeting of HBx, but this is now considered unlikely. Moreover, using these candidates requires repeated dosing, sometimes combined with other treatments, such as pegylated IFN, usually with concurrent administration of NAs as a backbone (e.g., ARB-1740).

Historically, ARC-520 emerged as the pioneer RNAi therapeutic capable of diminishing all RNA transcripts originating from cccDNA, marking its entry into clinical trials. That is, the Heparc-2001, -2002, and -2003 trials showcased the safety and efficacy of ARC-520 in attenuating HBsAg levels, though with certain limitations. Uniformly across the ARC-520 trials, a correlation exists between dosage and HBsAg response, with the responses from the low-dose cohorts failing to achieve statistical significance. The lowest HBsAg levels in both low- and high-dose treatment groups were observed on days 71 and 99, respectively. After this, a consistent elevation in HBsAg levels was observed, with the levels in the low-dose cohort approximating those observed in the placebo cohort just 30 days after the final administration [138]. In addition, the high-dose group exhibited notable reductions in HBsAg even 70 days after hitting their lowest level [138]. However, it is worth noting that total HBsAg reductions were not reached and that the HBsAg reductions were considerably less pronounced in HBeAg-negative individuals. This discrepancy may be due to ARC-520’s predilection for targeting cccDNA-derived pgRNA over integrated HBs, which serves as the primary source of HBsAg in older HBeAg-negative patients. Consequently, ARC-521 was formulated to improve ARC-520 action and target both integrated HBs and cccDNA [139]. The resultant decrease in HBsAg and HBV DNA was substantial, and the therapeutic agents were generally well-tolerated, with no adverse infusion reactions and no significant elevations in ALT [139,140]. It was also suggested that the failure for ARC-520 was due to its lack of targeting HBx; this was formally disproven by the failure of ARB-1467 and RG6004 (RO 7062931), which included HBx triggers inside HBV DNA integration breakpoints. Notably, the development works of ARC-520 and ARC-521 ceased following reports of mortality induced in non-human primates and due to substantial advances in developing other more promising RNAi therapeutics.

Similarly to previous candidates, ARB-1467 has exhibited substantial efficacy in reducing HBsAg levels. This candidate consists of a mixture of three triggers (two targeting the ORF of HBV S and one targeting HBV X) with a high-efficiency delivery to hepatocytes via lipid nanoparticle formulation (Table 1). The most significant reductions were evident among patients on stable nucleotide therapy, with several of the patients achieving exceptionally low HBsAg counts below 50 IU/mL. Nevertheless, scallop-like responses in HBsAg levels were observed in some patients, with rebounds in HBV RNA and HBcrAg levels during the course of treatment [141]. Concurrently, ARB-1467 maintained an admirable safety profile with negligible adverse events and consistently normal ALT levels [142].

Drawing parallels, other candidates have also revealed amplified efficacies when synergized with additional therapeutic agents. For instance, during its preclinical phase in mice, BB-103, when paired with Peginterferon or entecavir, achieved a remarkable 4-log reduction in HBV DNA and a 2-log decrement in HBsAg levels [143]. Similarly, ARB-1740, while independently showcasing reductions in multiple HBV markers such as BsAg, HBeAg, HBcAg, and HBV RNA, exhibited heightened efficacy when combined with a capsid inhibitor or pegylated interferon-alpha [144]. A recent clinical trial registered under the identifier NCT04225715 is designed to assess the safety and efficacy of various combination therapies. This study comprises nine distinct testing groups, each characterized by a unique combination of therapeutic agents. Notably, seven of these testing groups incorporate siRNA RO7445482 as one of the constituent therapeutics. Additionally, the groups encompass Nucleos(t)ide, CpAM, TLR7, PEG-IFN, and PD-L1 LNA therapeutic agents. While the primary results of this trial are anticipated in 2024, it is worth noting that this investigation holds promise and underscores the potential of combinatorial therapeutic strategies in augmenting the effectiveness of treatments for HBV. In a prior examination of the triple therapy consisting of JNJ-3989 RNAi, CpAM, and NAs, a notable outcome was achieved. Specifically, among a cohort of 12 individuals afflicted with chronic HBV, the treatment regimen yielded a substantial mean reduction of 1.7 logs in HBsAg levels by day 113. Furthermore, marked suppression was observed in various other viral markers, including HBV DNA, RNA, and HBcrAg. Importantly, the administration of this combinatorial therapy was well-tolerated overall, with no reports of severe adverse events. Mild elevations in ALT levels were observed in five patients as the primary side effect [145]. Significantly, the preclinical investigation has revealed an intriguing potential synergy between CRISPR and RNAi. Notably, this combined approach demonstrated enhanced outcomes in inhibiting HBV replication and eradicating HBV cccDNA when juxtaposed with the individual employment of CRISPR or RNAi techniques [146]. These compelling findings offer hope to advance our understanding of combinatorial therapeutic strategies and their potential to revolutionize the management of HBV infections.

A paramount consideration in the realm of HBV therapeutics is the sustained efficacy of treatments over extended periods. While numerous therapeutics exhibit robust short-term effectiveness, their potency often diminishes over time, underscoring the need for durable and consistent long-term solutions. The time-dependent efficacy of ARC-520 was shown by a mathematical model, indicating an initial reduction efficacy for HBsAg and HBeAg of up to 96% on day 1, which gradually tapered to around 50% over the subsequent 1.5–3 months. The model predicted a sustained efficacy exceeding 99.8% when ARC-520 was co-administered with entecavir [147], proving again the synergized activity of HBV therapeutics. On the other hand, the GalNAc–conjugated candidates, VIR-2218 and JNJ-3989, have demonstrated sustained therapeutic responses. VIR-2218 ensured a persistent decline in HBsAg for 28 weeks post-treatment across both HBeAg- and HBeAg+ patient groups and at varying dosage levels [148]. JNJ-3989 reported even more favorable outcomes, with 56% of patients maintaining a reduced HBsAg response nine months after treatment [149]. Notably, both these candidates have commendable safety profiles. Adverse events were infrequent; crucially, none were directly attributed to the respective therapeutic agents.

Therapeutics employing RNA interference appear promising for future hepatitis B treatment. Several candidates entered the clinical testing phase, with some already in advanced phase 3 testing, i.e., GSK3389404 (bepirovirsen, NCT05630820). This therapeutic targets all HBV mRNAs and was designed to utilize a binding site that is highly conserved across various HBV genotypes, where single-point mutations can potentially alter the polymerase and X protein [150,151]. However, according to one hypothesis, the activity of this drug may not be primarily due to the antisense effect but through immune stimulation of Toll-like receptor 9 (due to recognition of class II CpG motif) [152], although the role of Toll-like receptor 8 was also suggested [151]. It is crucial to fully understand the mechanism of action of such agents.

One should note that as long as such therapeutics may provide novel options in HBV treatment, they are still far from optimal, with subcutaneous or intravenous administration requiring repeated dosing over weeks or months of treatment. This may be similar to the era of interferon treatments in hepatitis C, which, apart from tolerance issues, were challenging due to their regime. The field was revolutionized with the subsequent introduction of direct-acting antivirals that are available orally [153,154]. Obviously, there are several differences between the hepatitis C virus and HBV, a major one being the ability of the latter to form cccDNA, preventing a similar treatment approach. However, one should note that there is some progress in the development of oral delivery for therapeutics based on RNA interference, e.g., for cancer treatment [155,156], indicating that in the future, similar therapeutics aiming to treat hepatitis B may eventually become available. However, it is crucial to ensure that these therapeutics will not be prone to resistance. Selection of escape mutants, novel or pre-existing, under therapeutics utilizing RNA interference in treatments of HCV, HIV, or poliovirus infections has been experimentally shown [157,158,159,160]. Therefore, it is essential to understand the risk of the emergence of escape mutants under the HBV treatments utilizing RNAi. Potential selection of resistance may lead to rebound and increased challenges in HBV treatment. However, one should note that the small size of the HBV genome likely limits its degree of redundancy, potentially dismissing the issues of resistance. Moreover, the potential combination of RNAi therapeutics with other, more conventional treatments may be a potential avenue to increase an overall antiviral effect and decrease the emergence of resistance to NA [161].

An additional concern is that in the majority of phase 2 trials of anti-HBV combination therapies, including siRNA or ASO ± CAM ± NUC ± PEG-IFN, there is a high proportion of patients experiencing viral rebound off-therapy. Currently, functional cure using novel antivirals is obtainable mainly in patients with low HBsAg levels, and therefore in the immune control phase. This shows that the lack of potent components restoring immune exhaustion in novel anti-HBV combos may hinder their long-term efficacy. Importantly, currently, the available animal models of HBV infection have several limitations, which do not allow them to fully and accurately simulate HBV infections occurring in humans. This may explain the discrepancies between the effects seen in the preclinical phase compared to the results of clinical trials of candidates based on RNA interference [162,163]. This results in the discontinuation of further testing of some candidates despite the promising *in vivo* observations. One of the recent examples of such a case includes ALG-125755, a subcutaneous GalNAc-conjugated siRNA, for which a single dose in mice led to HBsAg reduction by 1.5 log IU/mL sustained for six weeks [164,165], but clinical trials of which were eventually discontinued due to inconclusive efficacy results [166].

**Table 1:** HBV therapeutics based on RNA interference introduced to clinical phases of testing, results of which have been published.

Candidate	Phase	Targets	Key Findings	Disadvantages and Adverse Events	References
ALG-125755	1	Single trigger within the ORF of HBV S	(1) Well tolerated with good safety profile of single dose in BeAg negative individuals (2) Dose-dependent reductions in HBsAg over 90 days after a single dose	(1) Discontinued due to inconclusive efficacy results in human trial	[166,167,168]
ARC-520	2	Two triggers within the ORF of HBV X protein	(1) Prolonged HBsAg reduction (2) Targets all cccDNA-derived viral transcripts, preventing protein translation, reducing viral antigens and HBV DNA	(1) Limited effect on integrated HBV DNA (2) Differential response in patient populations (3) Terminated after mortality induced by the excipient in non-human primates was reported (4) Dosing in these studies was very high, but at normal dosing, no HBsAg response was observed	[81,138,164,169]
ARB-1467	2a	Three triggers targeting the ORF of HBV S and HBV X domains	(1) Single and multiple doses induced HBsAg decline independent of HBeAg status (2) HBcrAg and HBsAg reductions were observed following multiple doses. (3) No serious adverse events; no increase in ALT levels		[141,142,170]
AB-729	2	HBV X	(1) Targets both sources of HBsAg (cccDNA-derived and integrated HBV DNA) (2) Significant decline of HBsAg (3) Well tolerated alone and in combination with IFN	(1) The decrease in total HBsAg clearly correlated with declines over time in M-HBsAg and L-HBsAg and not with S-HBsAg	[171,172]
VIR-2218	2	One trigger targeting the ORF of X protein	(1) HBsAg decline persisting for 28 weeks after treatment (2) Reductions in HBsAg in HBeAg− and HBeAg+ patients across all dose levels (3) Well tolerated, no significant increase in ALT level	(1) Differential patterns of decline suggest that early responses (<8 weeks) in HBsAg may not predict the final magnitude of decline.	[148,173]
JNJ-3989	2	HBV X and HBV S domains	(1) Targets both sources of HBsAg (cccDNA and integrated HBV DNA) (2) Regardless of HBe serostatus, three monthly doses combined with daily NUC treatment resulted in reduced HBsAg, HBV DNA, HBV RNA, and HBcAg levels (3) 56% of patients achieved a sustained HBsAg response 9 months after treatment (4) Well tolerated, with mild-to-moderate adverse events		[149,174,175,176]
ARB-1740	1a/b	3 triggers that target three highly conserved regions of the HBV genome	(1) HBV RNA reduction, leading to inhibition of HBsAg, HBeAg, and HBcAg (2) Synergistic activity against HBV relaxed circular DNA when used in combination with a capsid inhibitor and/or pegylated interferon-alpha (3) May have therapeutic benefits for hepatitis delta patients	(1) Suspended due to transferring the liver-targeted delivery system from liposomes to GalNAc–siRNA conjugates.	[144,177]
ARC-521	1	A conserved sequence in HBV X and S ORFs	(1) Targets cccDNA and integrated DNA (2) Potent reductions in HBsAg and HBV DNA (3) Silenced all HBV gene products (4) Suppression of HBeAg and HBsAg suppression (5) Well tolerated in human participants	(1) Deaths in a non-clinical toxicology study in non-human primates (related to drug EX1 delivery vehicle)	[139,178]
GSK3228836 (bepirovirsen)	2b	All HBV mRNAs	(1) Sustained HBsAg and HBV DNA loss in 9 to 10% of participants after 300 mg injections weekly for 24 weeks	(1) HBsAg reduction only in patients with low HBsAg (<1000 IU/mL) at baseline (2) Increased levels of ALT (3) Injection-site reactions, pyrexia, and fatigue were common	[179,180]
GSK3389404 (bepirovirsen conjugated to GalNAc)	2a	Pregenomic and mRNA transcripts	(1) Dose-dependent reduction in HBsAg after 12 weeks of treatment (2) Reduction in HBsAg in HBeAg+ and HBeAg- patients (3) Good safety profile	(1) Only 5% had a >1.5-log reduction in HBsAg with no HBsAg seroclearance achieved	[181,182]
RO7062931	1	A highly conserved sequence in the shared 3′ region	(1) Dose-dependent declines in HBsAg demonstrated target engagement (2) Good safety profile and tolerance	(1) Suboptimal magnitude of HBsAg reduction (2) Further development halted	[183]
RG6346	1	HBV S domain	(1) Four monthly doses resulted in substantial and durable reductions in HBsAg levels lasting up to 1 year following the last dose (2) Well-tolerated, mild, local adverse reactions		[184]

## 4. Conclusions

Treatment of chronic HBV infections has improved over the years but remains far from optimal, with long-awaited breakthroughs that would improve functional therapy or offer a cure. The biotechnological approach to HBV has been gaining interest with time, development, and improvement of various tools, which reveal high potential in viral treatment. However, whether they become a game-changer in managing HBV infections is yet to be seen. Gene-editing tools aiming to eradicate HBV from chronically infected patients are yet to be introduced in clinical trials, with high potential for CRISPR-based systems and a need for long-term safety monitoring due to potential off-targets. Even though these tools may never be authorized for HBV eradication, the experience gained while studying them may be beneficial in developing potential treatments for other viral infections. In turn, a number of candidates based on RNAi, which may offer novel opportunities for functional cure, have already been introduced to clinical trials. However, larger and longer trials are required to assess their efficacy and safety. Importantly, the gene-editing tools and RNA interference presented in this review are not the only novel HBV treatment tools under development and study. Firstly, there is a substantial interest in using nucleic acid polymers as antivirals to achieve functional cures in patients with chronic viral infection, with some candidates revealing promising results in the clinical phase of testing [185,186,187,188,189,190,191,192,193]. Secondly, there is an interest in seeking therapies targeting epigenetic silencing of the cccDNA to inhibit viral transcription, e.g., by inhibiting SIRT2 deacetylase or NAD(P)H:quinone oxidoreductase 1, or by preventing erasing of epigenetic marks by lysine demethylase 5 [194,195,196,197]. However, considering that prevention is always superior to treatment, it is essential to pursue global efforts in HBV vaccination.

## Data Availability

Not applicable.

## Supplementary Materials

The following supporting information can be downloaded at: https://www.mdpi.com/article/10.3390/v15122395/s1, Table S1: The summary of studies exploring the use of Zinc Finger Proteins (ZFP) including Zinc Finger Nucleases (ZFN) and Zing Finger Antiviral Proteins (ZAP) in the treatment of HBV infection [198]; Table S2: The summary of studies exploring the use of transcription activator-like effector nucleases (TALEN) in the treatment of HBV infection; Table S3: The summary of studies exploring the use of clustered regularly interspaced palindromic repeat-Cas system (CRISPR/Cas) in the treatment of HBV infection [199,200,201].
