# Supplementary material for: Gene-Editing and RNA Interference in Treating Hepatitis B: A Review
Source: Viruses. 2023 Dec 8;15(12):2395. doi: 10.3390/v15122395 (PMC10747710; doi:10.3390/v15122395)
Supplement: Supplementary file 1 [file viruses-15-02395-s001.zip › viruses-2714166-supplementary.pdf]

Supplementary Tables (1-3) for
"Biotechnological Approach in Treating Hepatitis B: A Review"
by Nadiia Kasianchuk, Krystyna Dobrowolska, Sofiia Harkava, Andreea Bretcan, Dorota Zarębska-Michaluk, Jerzy Jaroszewicz, Robert Flisiak, and Piotr Rzymski

Supplementary Table S1. The summary of studies exploring the use of Zinc Finger Proteins (ZFP) including Zinc Finger Nucleases (ZFN) and Zing Finger Antiviral Proteins (ZAP) in the treatment of HBV infection.

Year	Experimental model	Targets	Delivery method	Effectiveness	Off-targets and other limitations	Reference
2008	*In vitro:* Longhorn male hepatoma cells infected with duck HBV	Enhancer region	ZFPs were designed with flanking XhoI and SpeI restriction endonuclease sites, and zinc fingers were linked in tandem by the canonical TGEKP linker. Cell were co-transfected with pDHBV1.3 and pcDNA3.1(+) or pcDNA3.1(+)-ZFPa, -ZFPb, or -ZFP1	1) ZFPs significantly reduced viral pregenomic RNA production (by 32-42%). 2) ZFPs caused a significant reduction in the expression of viral core and surface proteins and the production of infectious viral particles.	- Off-targets not detected - No effect on cellular protein synthesis	[1]
2010	*In vitro:* Huh7, HEK-293	Upstream of the common polyadenylation site of the four overlapping viral mRNAs near the N-terminus of the core protein	Vector (cytomegalovirus and T7 promoters. ZNFs were fused to the codon-optimized version of FokI.	1) Transfection efficiency ranged from 35-46%. 2) 8/18 of ZFNs cleaved their homodimeric targets. 3) The ZFNs 6L and 6R together cleaved their heterodimeric target, but not when used individually. 4) A 29% reduction in the levels of pregenomic RNA was observed. 5) Frameshift mutations were found in 13/16 sequences, potentially leading to premature stop codons and significant truncations of the essential viral protein. 6) A significant portion of the cleaved target DNA remained linear or was misrepaired	- Off-targets not detected - Cytotoxicity	[2]
2013	*In vitro:* HepG2, Huh7, HEK-293T, HepDES19	Within the 100-nucleotide-long terminal redundant region in the viral RNA genome	Plasmids<br><u>rN-ZAP</u>: Expressed the N-terminal 254 a.a. zinc-finger motif domain of rat ZAP<br><u>hZAP-L</u>: Full-length human ZAP isomer<br><u>hZAP-S</u>: Full-length human ZAP isomer<br><u>hN-ZAP</u>: Expressed the N-terminal 254 a.a. of human ZAP, cloned into pcDNA4<br><u>Mutant hZAP-S</u>: Contained disrupted individual or all four putative zinc finger motifs in hZAP-S	1) ZAP overexpression significantly reduced HBV DNA by decreasing viral pgRNA levels.<br>2) Liver-specific host factors were not essential for ZAP's antiviral function.<br>3) ZAP primarily accelerated HBV RNA decay posttranscriptionally in the nucleus,<br>4) ZAP's antiviral activity against HBV required its zinc finger motifs for binding to HBV RNA.<br>5) ZAP worked alongside other interferon-stimulated genes for maximal antiviral protection.	- Off targets not reported - Potential effect on host gene expression	[3]

			DN-IRF3: Dominant-negative IRF3<br>DN-IκBα: Dominant negative IκB-alpha			
2014	*In vitro*<br>HEK-293T HepAD38	ZFN1: ORF P/X<br>ZFN2: ORF P/C<br>ZFN3: ORF P	Plasmid transfection<br>or self-complementary adeno-associated virus (scAAV)	1) Plasmid transfection: ZFN2: the highest target mutagenesis rate (34%). ZFN3: highest (sustained) HBV replication suppression rate<br>2) scAAV: ZFN2: the highest target mutation rate (43%). ZFN3: highest (sustained) HBV replication suppression rate.	- Minimal off-target mutagenesis<br>- Off-target in other genome regions not analyzed<br>- Cytotoxicity (ZFN2)	[4]
2015	*In vivo:* transgenic mice	Terminal redundant region (nt 1820–1918) of HBV pgRNA..	ZAP transgenic mouse: microinjection of pEFmZAPmyc into the fertilized egg nucleus of ICR mouse<br><br>ZAP transgenic mouse transfected pHBV4.1 was termed the ZAP-ICR/HBV mouse as the experimental group, and ICR mouse transfected pHBV4.1 was termed the ICR/HBV mouse as the control group.	1) ZAP transgenic mice had HBV 3.5 kb and 2.4/2.1 kb mRNA levels decreased by 8 and 21%, respectively.<br>2) HBV RC or HBV SS DNA levels in ZAP transgenic mice decreased significantly, with an 82% decrease in HBV DNA replicative intermediates.<br>3) ZAP transgenic mice had a few HBsAg-positive and HBcAg-positive cells.<br>4) ZAP-mediated HBV RNA reduction was due to transcriptional inhibition but possibly due to accelerating HBV RNA decay.<br>5) ZAP decreased HBV RNA, HBV DNA replication intermediates, viral protein expression	Off-targets not reported	[5]
2018	*In vitro*: HepG2, HepG2.2.15<br>*In vivo:* transgenic mice	HBV DNA EnhI	The N-terminal ZF domain was fused to KRAB repression domains from the human ZFP 10 gene.<br><br>HepG2.2.15: transfection with pcDNA3.1-ATF, pcDNA-nls-KRAB-flag, pcDNA-nls-ZFP-flag, pcDNA-ATF flag<br>Mice: injection of pcDNA 3.1(+), pcDNA-nls-KRAB-flag, pcDNA-ATF or pcDNA-nls-ZFP-flag	1) HBV mRNA levels were reduced by 63% with pcDNA3.1-ATF and 49% with pcDNA3.1(+)-nls-ZFP-flag, while pcDNA3.1(+)-nls-KRAB-flag had a minor effect.<br>2) pcDNA3.1-ATF inhibited HBeAg secretion *in vitro* and *in vivo* but did not affect HBsAg<br>3) Both pcDNA3.1-ATF and pcDNA3.1(+)-nls-ZFP-flag suppressed HBV core and x protein *in vitro*.<br>4) ATF significantly inhibited HBV replication *in vitro* and *in vivo*.<br>5) HBV EnhI-specific ATF significantly inhibited HBV transcription.<br>6) pcDNA3.1-ATF reduced HBV replication by 68% compared to pcDNA3.1(+)-nls-KRAB-Flag.	Off-targets not reported<br>- No cytotoxicity	[6]
2019	*In vitro*: HepG2	Unspecified	Cotransfection with a replication-competent HBV DNA plasmid (pHBV1.3x) and plasmids expressing individual ZAP isoforms differing in length	1) ZAPs inhibited HBV by accelerating the decay of HBV pregenomic RNA.<br>2) All ZAPs significantly inhibited the production of HBeAg and HBsAg.<br>3) Longer ZAP isoforms were more effective	- Off-targets not reported<br>- Cytotoxicity not reported	[7]

Supplementary Table S2. The summary of studies exploring the use of transcription activator-like effector nucleases (TALEN) in the treatment of HBV infection.

Year	Experimental model	Targets	Delivery method	Effectiveness	Off-targets and other limitations	Reference
2013	*In vitro*: Huh7, HepG2.2.15<br><br>*In vivo*: murine hydrodynamic model	S/P ORF C/P ORF P ORF S/pol, C/pol and pol ORFs of the HBV genome	Cotransfection with HBV replication-competent plasmid and plasmids expressing TALEN<br>*In vivo*: bolus injection with HBV target DNA (pCH-9/3091) and of mock pUC118 or 16 μg of pairs of left and right TALEN-expressing plasmids.	1) *In vitro* effects:<br>- S TALEN: HBsAg reduction (by 70% under hypothermic conditions).<br>- C TALEN: HBcAg reduction.<br>- P1 TALEN: HBsAg reduction.<br>- P2 TALEN: No significant HBsAg reduction.<br>2) *In vivo* effects:<br>- S TALEN: HBsAg knockdown in serum, viral particle equivalents reduction (~70%), targeted disruption of HBV sequences in the liver (58-87%).<br>- C TALEN: Viral particle equivalents reduction (~70%), HBcAg decreased in hepatocytes, targeted disruption of HBV sequences in the liver (58-87%).	- Potential off-target sites identified for each TALEN at distant locations.<br>- No *in vivo* toxicity	[8]
2014	*In vitro*:: Huh7<br><br>*In vivo*: C3H/HeN mice	L1/R1: ORF P<br>L2/R2: C ORF L3/R3: C ORF	Plasmid transfection using FuGene-HD	*1) In vitro effects:*<br>- TALENs L1/R1 reduced HBeAg and HBsAg production, decreased levels of pgRNA (20–50%) and cccDNA (10–20%).<br>- TALENs L2/R2 reduced in HBeAg, HBsAg, HBcAg expression, decreased levels of pgRNA (20–50%), a drastic decrease in cccDNA levels (twofold)<br>- TALENs L3/R3 did not show a significant decrease in HBeAg and HBsAg production.<br>*2) In vivo effects:*<br>- TALENs L2/R2 reduced serum HBeAg, HBsAg, HBV DNA, liver HBV pgRNA, and liver core proteins.	- Potential off-target sites ruled out<br>- No *in vitro* and *in vivo* cytotoxicity	[9]
2016	*In vitro*:: Huh7 and HepG.2.2.15	Core (C-TALEN) and surface (C-TALEN) open reading frames of HBV	Plasmid transfection using polyethylenimine	1) S-TALENs: a significant decrease in HBsAg secretion (when combined with pri-miR-31/5/8/9)<br>2) C-TALENs: limited impact (alone); increased inhibition of HBsAg when combined with pri-miR elements or artificial RNA interference.<br>3) Donor sequences with shorter flanking arms enhanced antiviral efficiency in transfected Huh7 cells.	- Not reported	[10]
2021	*In vitro*:: Huh7, HEK293 and HepG2.2.15	ORF C<br>ORF S	Plasmid transfection using lipofectamine 3000	1) *In vitro* effects:<br>- All TALENs exhibited site-specific targeted mutagenesis with no statistically significant difference in cleavage efficacy when using 2nd or 3rd generation.	2nd-gen TALENs: Off-target mutations in	[11]

	In vitro: female NMRI mice			- TALENs S: All generations decreased HBsAg levels<br>- TALENs C: 1st generation did not affect HBsAg expression; 2nd generation considerably reduced HBsAg levels (50%), 3rd generation (Sharkley): did not affect HBsAg expression; significant inhibition of HBV, but lower than in 1 gen TALENs<br>2) *In vivo* effects:<br>- All TALENs, whether targeting the core or the surface ORF, affected suppression of circulating VPEs<br>- TALENs S: 1st generation decreased circulating VPEs; 2nd generation silenced HBsAg (>90%), decreased in circulating VPEs (80-90%); 3rd generation (Sharkley) silenced HBsAg levels (40–60%), decreased circulating VPEs (80-90%)<br>- TALENs C: 1st generation decreased HBsAg and circulating VPEs; 2nd generation decreased HBsAg and circulating VPEs (80-90%); 3rd generation (Sharkley): decreased HBsAg and circulating VPEs (80-90%).	murine Pah gene. No hepatotoxicity observed in mice.	

Supplementary Table S3. The summary of studies exploring the use of clustered regularly interspaced palindromic repeat-Cas system (CRISPR/Cas) in the treatment of HBV infection.

Year	Experimental model	Targets	Delivery method	Effectiveness	Off-targets and other limitations	Reference
2014	*In vitro*:: Huh7<br>*In vivo:* C57BL/6 mouse model	8 gRNAs S1, PS1, PS2, PS3, P1, XCp, eE, PCE	Lipid nanoparticle (Lipofectamine 2000, Lipofectamine 3000)<br><br>Hydrodynamic injection	1) *In vitro* effects:<br>- P1 gRNA: significant suppression of HBV protein (70%); with Cas9-expression cassette - 96% suppression of intracellular surface proteins;<br>- S1 gRNA: suppression of HBV protein (40%); with Cas9-expression cassette - 90% suppression of intracellular surface proteins;<br>- XCp gRNA: suppression of HBV protein (30%); with Cas9-expression cassette - 82% suppression of intracellular surface proteins;<br>- PS2 gRNA: suppression of HBV protein (20%); with Cas9-expression cassette -77% suppression of intracellular surface proteins<br>2) *In vivo* effects:<br>- P1 gRNA: significant serum HBsAg reduction; 5% intrahepatic disruption XCp gRNA: significant serum HBsAg reduction; 4% intrahepatic disruption	- Off-targets not reported<br>- No *in vitro* and *in vivo* toxicity	[12]
2015	*In vitro*:: HCC HepG2.2.15, HepG2-H1.3	S and X ORFs	Lipid Nanoparticle (Lipofectamine 2000)<br><br>Lentiviral Vector	1) Inhibition of HBV particle release<br>2) Impaired HBsAg release: Likely targeted viral cccDNA in *de novo* infections.<br>3) HBV Genome Editing: 44% to 89% of sequences exhibited indel modifications.	- Off-target not expected	[13]
2015	*In vitro*:: HepG2, HepG2.2.15, 293T cells<br><br>*In vitro*: NRG mice	S, P, C, X ORFs of HBV genome	Lentivirus vector<br><br>Lipid Nanoparticle (TransIT-2020)	*1) In vitro* effects:<br>- sgRNAs 17 and 21 (sg17 and sg21) decreased HBV pgRNA levels and HBsAg production.<br>- Combination of sg17 and sg21 showed stronger reductions in HBsAg and HBV 3.5kb RNA compared to the single guide RNAs.<br>- In HepG2.2.15, Cas9/sgRNAs induced significant suppression of HBV DNA release (77-95% decrease across different sgRNAs), HBeAg secretion, viral mRNA production, and cccDNA levels.<br>2) *In vivo* effects:	- Off-target not detected<br>- Toxicity not reported	[14]

				- Cas9 and sg21 showed a progressive suppression of HBV expression in mice, with a 4-fold decrease in viremia at day 4 post-injection.		
2016	*In vitro*:: Huh7, HepG2<br><br>*In vitro*: M-TgHBV mouse model	ORF S<br>ORF X<br><br>Homologous sequences from genotype A to H of HBV.	Lipid nanoparticle (Lipofectamine 3000)<br><br>Hydrodynamic injection	1) *In vitro* effects:<br>- pCas9-2 (ORF X): Significant reduction in HBcAg (66%), HBsAg (50%), HBeAg (48%), and cccDNA.<br>- pCas9-1 (ORF S): Reduction in HBcAg (45%), HBsAg (50%), HBeAg (20%) secretion<br>2) *In vivo* effects:<br>- pCas9-2: Significant decrease in liver HBcAg and serum HBsAg levels 10 days post-injection.<br>- pCas9-1: Decrease in serum HBsAg.	- Off-target not reported<br>- No toxicity	[15]
2017	*In vitro*:: HepG2.A64	ORF S	Lipid Nanoparticle (Lipofectamine LTX)	1) gRNA-91: HBeAg reduction (83.13%); HBsAg reduction (87.38%); HBV DNA reduction (91.72%)<br>2) gRNA-69: HBeAg reduction (80.53%); HBsAg reduction (86.49%); the most significant DSBs of the integrated HBV genome; HBV DNA reduction (89.21%)ж HBV cccDNA was <500 copies/10^6 cells<br>3) gRNA-62: HBeAg reduction (70.71%); HBsAg reduction (80.07%); HBV DNA reduction (80.30%)<br>4) gRNA-60: HBeAg reduction (76.50%); HBsAg reduction (82.55%); HBV DNA reduction (86.95%)<br>5) gRNA-69-7: HBV DNA reduced to 420 ± 278 IU/mL; HBV cccDNA undetectable (<500 copies/10^6 cells); no detectable HBV cccDNA, HBV DNA, HBsAg, HBeAg for 10 months.	No off-target observed	[16]
2020	*In vitro*::HEK293, Huh7<br><br>*In vitro*: liver-humanized chimeric FRG mice	containing tandem copies of three HBV-specific sgRNA target sites (C1-C3-C6 and C7-C14-C16)	Lipid Nanoparticle (Lipofectamine 3000)<br><br>Cationic Polymer (Polyethylenimine)<br><br>Adeno-Associated Virus Vector (AAV-SaCas9)	1) Higher survival rate of human hepatocytes in AAV-SaCas9 treated mice compared to controls.<br>2) Observed HBV target site mutations in the liver of treated mice.<br>3) No significant reduction in serum HBV DNA post-treatment<br>4) Levels of cccDNA reduced in treated mice, although not statistically significant.<br>5) Mice with higher SaCas9 transfer into HBV+ cells showed increased gene editing.	- No off-target observed - AAV-SaCas9 therapy well-tolerated with no adverse effects	[17]
2020	*In vitro*:: HEK293T, HepG2.2.15, HepAD38, HepG2-NTCP-C4, Huh7	23 candidate target sequences, including 3 in the core, 9 in polymerase, 9 in the surface, and 2 in X ORFs	Lentiviral Vector<br><br>Lipid Nanoparticle (Lipofectamine 3000)	1) 14/23 gRNAs showed base-editing efficiency ≥50% for polymerase and surface ORFs.<br>2) gRNAs + BE4: Substantial HBsAg suppression in HepG2.2.15 cells.<br>3) Polymerase-targeting gRNAs: HBV DNA levels decreased by >60%.	- Minimal off-target mutations from base editing were. - On-target	[18]

				4) gRNAs like gP8 and gP9 led to significant HBsAg reduction.<br>5) gS3, gS7, gS8 targeting surface ORFs: significant decrease in HBV DNA levels	indels for gRNAs were between 0.5%-5%.<br>- WT Cas9 had higher levels of indels (>70%) than Cas9-BE	
2021	*In vitro*: HEK293T<br><br>*In vivo:* C57BL/6 mice;	X, C, preS1 and preS2 ORFs	Lipid Nanoparticle (Lipofectamine 2000)<br><br>Cationic Polymer (Neofect)<br><br>Adeno-Associated Virus Vector (AAV8)<br><br>Lentivirus vector	1) *In vitro* effects:<br>- All gRNAs of the reconstructed CRISPR/SaCas9 systems reduced average HBsAg and HBeAg levels by 25-85%.<br>- T2, T3, T6, and Tmix reduced HBsAg and HBeAg levels by more than half<br>- All gRNAs dramatically suppressed HBV replication and reduced the amount of rcccDNA.<br>- HBV RNA transcripts were stably reduced by T2, T3, and T6.<br>2) *In vivo* effects:<br>- HBV core protein expression in mouse liver and serum HBsAg levels were significantly inhibited after AAV8-delivered CRISPR/SaCas9 treatment. Significant inhibition of HBeAg observed with AAV8-T2.<br>- HBV DNA in the serum and HBV RNA in the liver were significantly reduced after T2, T6, or Tmix treatment.	- AAV8 showed clear liver tropism effects, reducing the possibility of off-target cleavage in other organs	[19]
2022	*In vitro*: HepG2-2.15	ORF X	Lipid Nanoparticle (Lipofectamine 2000)	1) Notable reductions in HBsAg and HBV cccDNA.<br>2) Suppressed tumorigenic properties like cell proliferation, migration, and invasion in HepG2.2.15 cells.<br>3) Altered key genes associated with EMT and cancer stemness.<br>4) In 3D models, reduced HBV replication and tumor spheroid formation after HBx knockdown.	- Minimum off-target mismatches<br>- Toxicity not reported	[20]

References

1. Zimmerman, K.A.; Fischer, K.P.; Joyce, M.A.; Tyrrell, D.L.J. Zinc Finger Proteins Designed to Specifically Target Duck Hepatitis B Virus Covalently Closed Circular DNA Inhibit Viral Transcription in Tissue Culture. *J. Virol.* **2008**, *82*, 8013–8021.
2. Cradick, T.J.; Keck, K.; Bradshaw, S.; Jamieson, A.C.; McCaffrey, A.P. Zinc-Finger Nucleases as a Novel Therapeutic Strategy for Targeting Hepatitis B Virus DNAs. *Mol. Ther.* **2010**, *18*, 947–954.
3. Mao, R.; Nie, H.; Cai, D.; Zhang, J.; Liu, H.; Yan, R.; Cuconati, A.; Block, T.M.; Guo, J.-T.; Guo, H. Inhibition of Hepatitis B Virus Replication by the Host Zinc Finger Antiviral Protein. *PLoS Pathog.* **2013**, *9*, e1003494.
4. Weber, N.D.; Stone, D.; Sedlak, R.H.; De Silva Feelixge, H.S.; Roychoudhury, P.; Schiffer, J.T.; Aubert, M.; Jerome, K.R. AAV-Mediated Delivery of Zinc Finger Nucleases Targeting Hepatitis B Virus Inhibits Active Replication. *PLoS One* **2014**, *9*, e97579.
5. Chen, E.-Q.; Dai, J.; Bai, L.; Tang, H. The Efficacy of Zinc Finger Antiviral Protein against Hepatitis B Virus Transcription and Replication in Tansgenic Mouse Model. *Virol. J.* **2015**, *12*, 25.
6. Luo, W.; Wang, J.; Xu, D.; Bai, H.; Zhang, Y.; Zhang, Y.; Li, X. Engineered Zinc-Finger Transcription Factors Inhibit the Replication and Transcription of HBV in Vitro and in Vivo. *Int. J. Mol. Med.* **2018**, *41*, 2169–2176.
7. Li, M.M.H.; Aguilar, E.G.; Michailidis, E.; Pabon, J.; Park, P.; Wu, X.; de Jong, Y.P.; Schneider, W.M.; Molina, H.; Rice, C.M.; et al. Characterization of Novel Splice Variants of Zinc Finger Antiviral Protein (ZAP). *J. Virol.* **2019**, *93*, doi:10.1128/JVI.00715-19.
8. Bloom, K.; Ely, A.; Mussolino, C.; Cathomen, T.; Arbuthnot, P. Inactivation of Hepatitis B Virus Replication in Cultured Cells and in Vivo with Engineered Transcription Activator-like Effector Nucleases. *Mol. Ther.* **2013**, *21*, 1889–1897.
9. Chen, J.; Zhang, W.; Lin, J.; Wang, F.; Wu, M.; Chen, C.; Zheng, Y.; Peng, X.; Li, J.; Yuan, Z. An Efficient Antiviral Strategy for Targeting Hepatitis B Virus Genome Using Transcription Activator-like Effector Nucleases. *Mol. Ther.* **2014**, *22*, 303–311.
10. Dreyer, T.; Nicholson, S.; Ely, A.; Arbuthnot, P.; Bloom, K. Improved Antiviral Efficacy Using TALEN-Mediated Homology Directed Recombination to Introduce Artificial Primary MiRNAs into DNA of Hepatitis B Virus. *Biochem. Biophys. Res. Commun.* **2016**, *478*, 1563–1568.
11. Smith, T.; Singh, P.; Chmielewski, K.O.; Bloom, K.; Cathomen, T.; Arbuthnot, P.; Ely, A. Improved Specificity and Safety of Anti-Hepatitis B Virus TALENs Using Obligate Heterodimeric FokI Nuclease Domains. *Viruses* **2021**, *13*, 1344.
12. Lin, S.-R.; Yang, H.-C.; Kuo, Y.-T.; Liu, C.-J.; Yang, T.-Y.; Sung, K.-C.; Lin, Y.-Y.; Wang, H.-Y.; Wang, C.-C.; Shen, Y.-C.; et al. The CRISPR/Cas9 System Facilitates Clearance of the Intrahepatic HBV Templates in Vivo. *Mol. Ther. Nucleic Acids* **2014**, *3*, e186.
13. Karimova, M.; Beschorner, N.; Dammermann, W.; Chemnitz, J.; Indenbirken, D.; Bockmann, J.-H.; Grundhoff, A.; Lüth, S.; Buchholz, F.; Wiesch, J.S.Z.; et al. CRISPR/Cas9 Nickase-Mediated Disruption of Hepatitis B Virus Open Reading Frame S and X. *Sci. Rep.* **2015**, *5*, doi:10.1038/srep13734.
14. Ramanan, V.; Shlomai, A.; Cox, D.B.T.; Schwartz, R.E.; Michailidis, E.; Bhatta, A.; Scott, D.A.; Zhang, F.; Rice, C.M.; Bhatia, S.N. CRISPR/Cas9 Cleavage of Viral DNA Efficiently Suppresses Hepatitis B Virus. *Sci. Rep.* **2015**, *5*, 1–9.
15. Zhu, W.; Xie, K.; Xu, Y.; Wang, L.; Chen, K.; Zhang, L.; Fang, J. CRISPR/Cas9 Produces Anti-Hepatitis B Virus Effect in Hepatoma Cells and Transgenic Mouse. *Virus Res.* **2016**, *217*, 125–132.
16. Li, H.; Sheng, C.; Wang, S.; Yang, L.; Liang, Y.; Huang, Y.; Liu, H.; Li, P.; Yang, C.; Yang, X.; et al. Removal of Integrated Hepatitis B Virus DNA Using CRISPR-Cas9. *Front. Cell. Infect. Microbiol.* **2017**, *7*, doi:10.3389/fcimb.2017.00091.
17. Stone, D.; Long, K.R.; Loprieno, M.A.; De Silva Feelixge, H.S.; Kenkel, E.J.; Liley, R.M.; Rapp, S.; Roychoudhury, P.; Nguyen, T.; Stensland, L.; et al. CRISPR-Cas9 Gene Editing of Hepatitis B Virus in Chronically Infected Humanized Mice. *Mol. Ther. Methods Clin. Dev.* **2021**, *20*, 258–275.

18. Yang, Y.-C.; Chen, Y.-H.; Kao, J.-H.; Ching, C.; Liu, I.-J.; Wang, C.-C.; Tsai, C.-H.; Wu, F.-Y.; Liu, C.-J.; Chen, P.-J.; et al. Permanent Inactivation of HBV Genomes by CRISPR/Cas9-Mediated Non-Cleavage Base Editing. *Mol. Ther. Nucleic Acids* **2020**, *20*, 480–490.
19. Yan, K.; Feng, J.; Liu, X.; Wang, H.; Li, Q.; Li, J.; Xu, T.; Sajid, M.; Ullah, H.; Zhou, L.; et al. Inhibition of Hepatitis B Virus by AAV8-Derived CRISPR/SaCas9 Expressed from Liver-Specific Promoters. *Front. Microbiol.* **2021**, *12*, doi:10.3389/fmicb.2021.665184.
20. Rawal, P.; Tripathi, D.M.; Hemati, H.; Kumar, J.; Sarin, S.K.; Nain, V.; Kaur, S. Targeted HBx Gene Editing by CRISPR/Cas9 System Effectively Reduces Epithelial to Mesenchymal Transition and HBV Replication in Hepatoma Cells. *Research Square* 2022.
